# Prevalence of eyelid lesions in cutaneous leishmaniasis in Pakistan

**Published:** 2015

**Authors:** Tayyab Afghani, Hassan Mansoor, Sultan A Kiani, Mukhtar A Mirza

**Affiliations:** Professor and Head: Department of Orbit and Oculoplastics, Al-Shifa Trust Eye Hospital, Rawalpindi, Pakistan.; Registrar: Department of Orbit and Oculoplastics, Al-Shifa Trust Eye Hospital, Rawalpindi, Pakistan, hassan-mansoor@hotmail.com; Associate professor: Department of Orbit and Oculoplastics, Al-Shifa Trust Eye Hospital, Rawalpindi, Pakistan.; Medical superintendent: Mines Labor Welfare Hospital, Chakwal, Pakistan.

**Figure 1:**
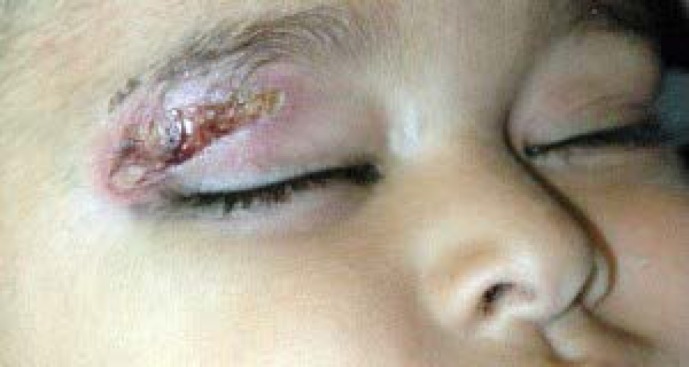
Right upper lid superficial ulcerative lesion with moderate ptosis (3 mm). Sultan Asif Kiani

Cutaneous leishmaniasis is endemic in over 80 countries of the world, encompassing almost five continents. According to the World Health Organization, leishmaniasis has an estimated incidence of twelve million worldwide.^2^ Leishmaniasis is a parasitic disease caused by a haemoflagellate Leishmania. It is transmitted to humans by the bite of female sand flies, of which there are 30 species. The epidemiology of cutaneous leishmaniasis is strongly correlated with the temporal and geographical distribution of the vector (the sand fly). The activity of the sand fly is in turn affected by both rainfall and temperature. Extensive land reclamation, the presence of infected rodents and unnaturally moist soil all lead to an increase in the density of the vector.

Cutaneous leishmaniasis is endemic in many regions of Pakistan and is spreading widely.^1^ This is probably due to the neighboring endemic belt of Afghanistan that has invaded the rural and urban areas of Pakistan. The Baluchistan Province and the Sindh Province have been declared endemic for the disease.

This study was carried out to measure the prevalence of eyelid lesions in cutaneous leishmaniasis in the central region of Punjab Province in Pakistan.

## Materials and methods

A cross-sectional study was conducted to measure the prevalence of eyelid lesions in cutaneous leishmaniasis in 925 patients with known cutaneous leishmaniasis and registered for treatment at Mines Labour Welfare Hospital in District Chacwai. The hospital is a referral centre for leishmaniasis and serves a population of approximately 5 million people in Distract Chacwai and adjoining districts. We examined the gender and age distribution of the patients with cutaneous leishmaniasis having eyelid lesions. We described cutaneous and eyelid lesions, measured visual acuity, and determined prevalence of the eyelid lesions in all 925 patients.

**Figure 2.**
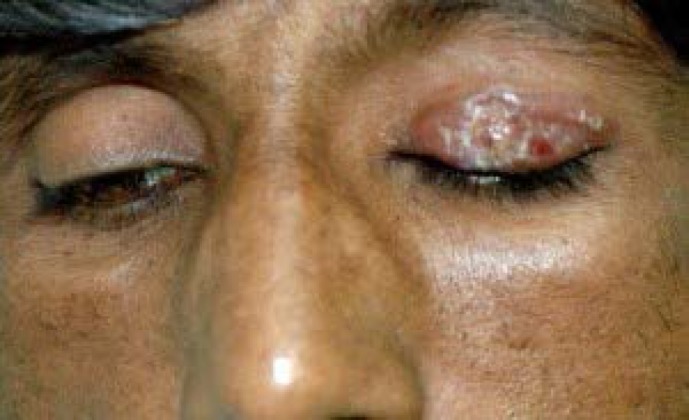
Left upper lid superficial ulcerative lesion with moderate ptosis (3mm). Sultan Asif Kiani

**Figure 3.**
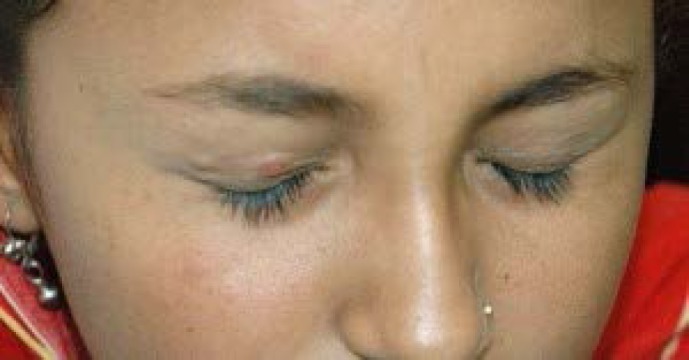
Right upper lid plaque-like lesion with moderate ptosis (3 mm). Sultan Asif Kiani

## Results

Out of the 925 patients, 395 were male (43%) and 530 were female (57%). The patients had 1,113 cutaneous lesions in total. Of these lesions, 202 were present on the face (18.14%), 22 on the forehead (1.97%), 8 on the eyelid (0.72%), 70 on the nose (6.28%), 97 on lips (8.71%), 73 On fingers (6.55%), 204 On hands (18.32%), 215 on arms (19.31%), 94 on legs (8.44%), 109 on feet (9.79%) and 19 on the trunk (1.70%). Only 8 patients had eyelid involvement, which accounts for a prevalence of 0.86%. Of the 8 patients who had eyelid involvement, 5 were male and 3 were female. The age of the patients with eyelid lesions varied from 1 year to 60 years. The mean age of the 8 patients with eyelid leishmaniasis was 25 years. The right upper lid was involved in 3 cases (37.5%), the right lower lid was involved in 1 case (12.5%) and the left upper lid was involved in 4 cases (50%). Two patients with eyelid involvement (see Figure 1 and Figure 2) had superficial ulcerative lesions (25%), three had nodulo-ulcerative lesions (37.5%), two had nodular lesions (25%) and one (Figure 3) had a plaque-like lesion (12.5%). Mild to moderate ptosis (2–3 mm) was present in 7 out of the 8 patients who had eyelid (upper lid) involvement. However, the visual acuity wasn't affected in the 7 adult patients who had eyelid involvement with cutaneous leishmaniasis. Visual acuity couldn't be assessed in the infant patient who had isolated upper eyelid involvement.

## Discussion

The fact that the upper eyelid was predominantly involved in our study indicates that eyelid leishmaniasis has a potential for causing visual impairment. This disability however can be reversed in adults by reconstruction, while in children; the risk of amblyopia is a concern. Therefore, timely intervention in children is mandatory. We did not come across any globe involvement by leishmaniasis, which is another potential cause of visual impairment. Other reported ocular and periocular presentations of leishmaniasis include lagophthalmos, lacrimal discharge, blepharoconjuctivitis, nodular episcleritis, scleromalacia, ulcerative interstitial keratitis, conjunctivitis, anterior uveitis, secondary glaucoma and retinopathy.^3^ These presentations were not seen in the current study.

For diagnosis of cutaneous leishmaniasis, slit skin smears and touch impression smears are done to detect the parasite. There is no serological test for cutaneous leishmaniasis.

The patients in the current study were given injections of intra-lesional sodium stibogluconate (undiluted), which is a pentavalent antimonial compound. Injections were given once weekly until the infection was resolved.

## Conclusion

It is believed that poverty, rural background, old buildings, hot climate, relaxation of the insecticide spray regime and poor rainfall are the main factors responsible for the recent outbreaks in Pakistan. Urgent precautionary measures should be undertaken and the physical conditions of the houses and buildings be upgraded. Patients living in these endemic areas should be educated about cutaneous leishmaniasis, sand fly life cycles and preventive measures. Eyelid lesions in cutaneous leishmaniasis can lead to permanent ocular or periocular deformities and should therefore be treated.
